# Immunological and Genomic Analysis Reveals Clinically Relevant Distinctions between Angiosarcoma Subgroups

**DOI:** 10.3390/cancers14235938

**Published:** 2022-11-30

**Authors:** Stefan G. van Ravensteijn, Yvonne M. H. Versleijen-Jonkers, Melissa H. S. Hillebrandt-Roeffen, Marije E. Weidema, Maikel J. L. Nederkoorn, Kalijn F. Bol, Mark A. J. Gorris, Kiek Verrijp, Leonie I. Kroeze, Tessa J. J. de Bitter, Richarda M. de Voer, Uta E. Flucke, Ingrid M. E. Desar

**Affiliations:** 1 Department of Medical Oncology, Radboud University Medical Centre, 6525 GA Nijmegen, The Netherlands; 2 Department of Tumor Immunology, Radboud University Medical Centre, 6525 GA Nijmegen, The Netherlands; 3 Department of Pathology, Radboud University Medical Centre, 6525 GA Nijmegen, The Netherlands; 4 Department of Human Genetics, Radboud University Medical Centre, 6525 GA Nijmegen, The Netherlands

**Keywords:** immunotherapy, sarcoma, tumor microenvironment, genetics, biomarker, angiosarcoma

## Abstract

**Simple Summary:**

Angiosarcomas (AS) are rare and aggressive soft tissue sarcomas that can be subdivided in de novo primary AS and secondary AS. They have a very poor prognosis and only limited treatment options are available. Immunotherapy is not registered as treatment for AS. Characterization of the immunological landscape of AS and combining this with genetic data enables possible identification of subgroups that are likely to benefit from immunotherapy based treatment strategies. We observed profound clinically relevant differences in a large group of primary and secondary AS. The T-cell infiltrated tumor microenvironment and frequent DNA Damage Response gene mutations, especially in secondary AS, warrant trials with immunotherapy for this subgroup.

**Abstract:**

Angiosarcomas (AS) are extremely rare and aggressive vascular malignancies subdivided in de novo primary AS (pAS) and secondary AS (sAS). We hypothesize that the combination of immunological and genomic profiles significantly differs between primary and secondary AS, with potential impact on treatment strategies and a role for immunotherapy. Tumor-infiltrating lymphocytes were analyzed using multiplex immunohistochemistry from 79 pAS and 178 sAS. Median cell density was significantly higher in sAS for CD3^+^ T-cells (*p* < 0.001), CD8^+^ cytotoxic T-cells (*p* = 0.033), CD4^+^ T-helper cells *(p* < 0.001) and FoxP3^+^ T-regulatory cells (*p* < 0.001). CD20^+^ B-cell density was comparable (*p* = 0.417). Comprehensive genomic profiling was performed in 25 pAS and 25 sAS. A (likely) pathogenic mutation was detected in 80% of pAS vs. 88% of sAS (*p* = 0.702). Amplifications were found in 15% of pAS vs. 84% of sAS (*p* < 0.001). DNA damage response (DDR) pathway mutations (*p* = 0.021) and MYC amplifications (*p* < 0.001) were predominantly seen in sAS. In conclusion we observed a clear and clinical relevant distinction in immune infiltration and genomic profiles between pAS and sAS. The T-cell infiltrated tumor microenvironment and frequent DDR gene mutations, especially in sAS, warrant clinical trials with immunotherapy.

## 1. Introduction

Angiosarcomas (AS) are rare malignant mesenchymal tumors with endothelial characteristics [1,2]. They comprise of primary (de novo) AS (pAS) which can develop at different anatomic sites with unknown etiology and secondary AS (sAS) which arise due to DNA damaging factors like ultra-violet (UV) light exposure, prior radiotherapy or chronic lymphedema (Stewart-Treves syndrome) [1,2,3]. 

Unfortunately, the risk of developing local recurrent or metastatic disease in AS exceeds 50%. Treatment for localized disease consists of surgery, either in combination with (neo)adjuvant chemo- or radiotherapy. In locally advanced or metastatic disease median survival is limited to 5–10 months and patients are primarily treated with palliative chemotherapy. The only approved non-cytotoxic drug used is the tyrosine kinase inhibitor pazopanib [4,5]. Overall survival has not improved the last 10 years, emphasizing the need for better treatments [6,7,8].

Immune checkpoint inhibition (ICI) has become the cornerstone in treatment of various malignant tumors except sarcomas [9,10]. Most sarcomas have a poorly immune-infiltrated tumor microenvironment (TME) and are therefore considered less responsive to ICI [11,12]. However, some case reports and small case series demonstrated promising responses in AS patients treated with ICI, especially in UV-associated AS (UV-AS) [13,14,15,16,17]. D’Angelo et al. reported a durable clinical response in 3/8 AS patients treated with a combination of nivolumab and bempegaldesleukin, a CD122-preferential interleukin-2 pathway agonist [18]. It remains however unclear which AS clinical subgroups may benefit from ICI treatment. 

So far, unambiguous biomarkers to predict response to ICI are lacking. Tumor mutational burden (TMB) and programmed cell death ligand 1 (PD-L1) are the most studied biomarkers. It is increasingly clear that they do not inequivalently predict ICI response [16,18,19,20,21,22]. In sarcomas the composition of immune cell subsets, especially B- and T-cells within the TME, might be more indicative [23]. Petitprez et al. reported that in sarcomas, a CD8^+^ T-cell signature and PD-1 expression resulted in favorable outcomes when B-cells were highly present [23]. D’Angelo et al. have shown that CD8^+^ T-cell infiltrates were correlated with an improved objective response rate after treatment with ICI in a very limited population of AS among others [18].

Due to the heterogeneity of AS, and the limited efficacy of the current generic therapeutic options, there is an urgent need for further characterization of their combined immunological and genomic landscape to detect subgroups that are likely to benefit from ICI based treatment strategies. These strategies could comprise ICI monotherapy and combinations boosting ICI response [24]. Until now, no data are available on the composition of immune cells in AS while comprehensive genetic analyses of AS are performed in limited patient numbers [25,26]. We aim to investigate the immune environment in a large group of AS clinical subgroups, in order to explore those that might benefit from ICI based treatment and combine this with additional genetic data.

## 2. Methods

### 2.1. Objectives

The primary objective is to detect differences in the combined immunological and genomic profiles of primary versus secondary angiosarcomas. The secondary objectives are to explore both the immunological and genomic profile of clinical AS subgroups.

### 2.2. Patients

We retrospectively collected data and samples from the primary tumor of patients diagnosed with AS in the Netherlands between 1989 and 2015 by a nationwide search through PALGA (Dutch nationwide network and registry of histo- and cytopathology) and an additional search through the Pathology database of the Radboudumc (2015–2019). Clinical data were received from the nationwide Netherlands Cancer Registry. Ethical approval for the study was obtained from the local certified Medical Ethics Committee (2016–2686). Patients were categorized in 2 main clusters; pAS and sAS and 8 clinical subgroups based on location of the tumor and origin of the AS. pAS subgroups were divided into: Heart, primary breast, skin that is not UV associated, soft tissue and visceral [8]. sAS subgroups are: Radiotherapy associated AS (RT-AS), Stewart Treves AS and UV-AS. AS of the sun exposed skin of the head and neck area were classified as UV-AS. All samples were histopathologically reviewed and representative tumor cores were selected.

### 2.3. Tumor Microenvironment

Tissue microarrays (TMAs) of formalin-fixed paraffin embedded (FFPE) AS tumor material from 257 patients were used for immune profiling. To correct for heterogeneity two 2 mm tumor cores per tumor were analyzed. Multiplex immunohistochemistry (mIHC) was performed on 4 µm thick tissue sections of TMAs by use of the Opal 7-color IHC kit and automated Bond RX stainer using primary antibodies against CD3, CD8, FoxP3, CD20 and CD56 to detect cytotoxic, regulatory and T-helper cells, B-cells and Natural Killer (NK) cells. An antibody against ERG, a transcription factor expressed in endothelial cells, served as tumor marker (clone EPR3864, Abcam, Cambridge, UK). Methods for panel optimization and validation were previously described [27,28]. Multispectral images were generated by scanning the slides with the Vectra^®^ Automated Quantitative Imaging System with software version 3.0.4 (PerkinElmer, Waltham, MA, USA). Data were analyzed using inform software (Akoya Biosciences, Marlborough, MA, USA), extended with in-house developed software for cell identification, phenotyping and localization of immune cell subsets [29].

### 2.4. Genomic Analysis

Sample selection for next generation sequencing (NGS) was based on an estimated percentage of tumor cells of at least 30% to ensure accurate analysis of microsatellite instability (MSI) and tumor mutational burden (TMB). Tumor material could not be older than 2005, and only primary located untreated samples were included for the genomic analysis. DNA was extracted from FFPE tissue, precipitated and quantified. Library preparation and sequencing was performed as described previously [30,31]. Coverage tables and a variant call file for single- and multiple-nucleotide variants, including number and percentage of variant alleles, were provided. Genomic variants were filtered by excluding the following: (1) variants not overlapping with exons and splice site regions except those in the TERT promoter region, (2) synonymous variants, unless located in a splice site region, (3) variants present with a frequency >0.1% in the control population represented in The Exome Aggregation Consortium (ExAC) version 0.2, and (4) variants with a variant allele frequency of <5%. Identified candidate variants were confirmed using the software Alamut visual version 2.13. Variants were manually analyzed and classified based on the predicted pathogenicity into 5 classes: class 1, not pathogenic; class 2, unlikely pathogenic; class 3, variant of unknown significance; class 4, likely pathogenic; and class 5, pathogenic. Class 4 and 5 variants were considered potentially clinically relevant and are referred to in this article as (likely) pathogenic. Interpretation of pathogenicity for variants in tumor suppressor genes (TSGs) was based on three prediction tools (sorting intolerant from tolerant (SIFT), Polyphen-2 and Align-Grantham Variation Grantham Deviation (Align-GVGD) and for both TSG and oncogenes on various knowledge-based tools (ClinVar, OncoKB, InterVar)). Therapeutic targeting of tumor suppressor genes generally requires inactivation of both gene copies. Therefore, for class 4/5 variants in tumor suppressor genes we evaluated whether these affect one or two alleles based on relative coverage and/or variant allele frequencies (VAF) of the variant and nearby single nucleotide polymorphisms (SNPs). Presence of gene amplification was analyzed as previously described on the basis of median coverage normalization [30]. A relative coverage ≥3 was considered gene amplification. The number of gene copies was estimated by using the relative coverage corrected for the percentage of tumor cells in the sample. TMB analysis was based on both synonymous and non-synonymous variants (total TMB). A cutoff value of 10 mutations/Mb (mut/Mb) was considered high TMB. Mutational signatures were investigated in all tumors with a TMB ≥10 mutations/Mb by use of the COSMIC mutational signature v3 [30,32].

### 2.5. Statistical Analysis

Median values and interquartile ranges (IQR) were used to describe continuous variables. Count and percent were used for categorical variables. To compare variables across groups, the Fisher exact test was used for categorical variables and Mann–Whitney U test for continuous variables. Values were considered significant with a *p* value < 0.05. Statistical analysis was performed using IBM SPSS statistics 25 and R (version 3.6.2).

## 3. Results

### 3.1. Higher Immune Infiltration in sAS

**Figure 1:**
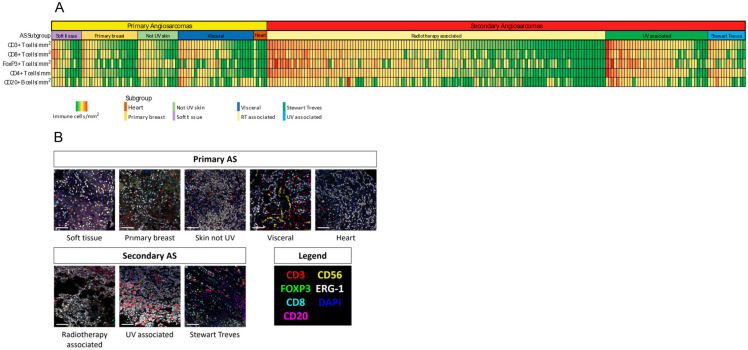
(**A**) Heat map identifying immune cell infiltrate densities per mm^2^ of CD3^+^, CD4^+^, CD8^+^, FoxP3^+^ T cells and CD20^+^ B-cells per AS subgroup. Each vertical bar representing an individual patient. (**B**) Example of Multiplex Immunohistochemistry images for all specific AS subgroups. Primary antibodies were used against CD3, CD8, FoxP3, CD20 and CD56 to detect cytotoxic, regulatory and T helper cells, B cells and Natural Killer (NK) cells. An antibody against ERG served as tumor marker.

Patient characteristics are depicted in Table 1. The median cell densities of lymphocyte subsets for pAS (*n* = 79) and sAS (*n* = 178) are shown in Table 2 and Figure 1. Median density was significantly higher in sAS for CD3^+^ T-cells, CD4^+^ T-helper cells, CD8^+^ cytotoxic T-cells and FoxP3^+^ T-regulatory cells. The median count of CD20^+^ B-cells was not significantly different between pAS and sAS. NK cells were rarely observed in both subgroups and never more than 30 cells/mm^2^ (median 0 cells/mm^2^).

Next, cell densities of specific AS subgroups were analyzed. A complete overview of the cell densities for individual subgroups is shown in Table 3. Within the sAS group, median cell density was significantly higher for all lymphocyte subsets in the UV-AS subgroup compared to the RT-AS group: CD3^+^ (*p* = 0.003), CD4^+^ (*p* = 0.004), CD8^+^ (*p* = 0.022) and FoxP3^+^ T-cells (*p* < 0.001) and CD20^+^ B-cells (*p* = 0.021). UV-AS also showed significantly higher densities across all lymphocyte subsets compared to de novo (not UV-associated) skin AS. Visceral AS showed the highest cell density of all pAS subgroups for CD3^+^ T-cells, CD4^+^ T-cells, CD8^+^ T-cells and CD20^+^ B-cells.

### 3.2. Genomic Landscape

**Figure 2:**
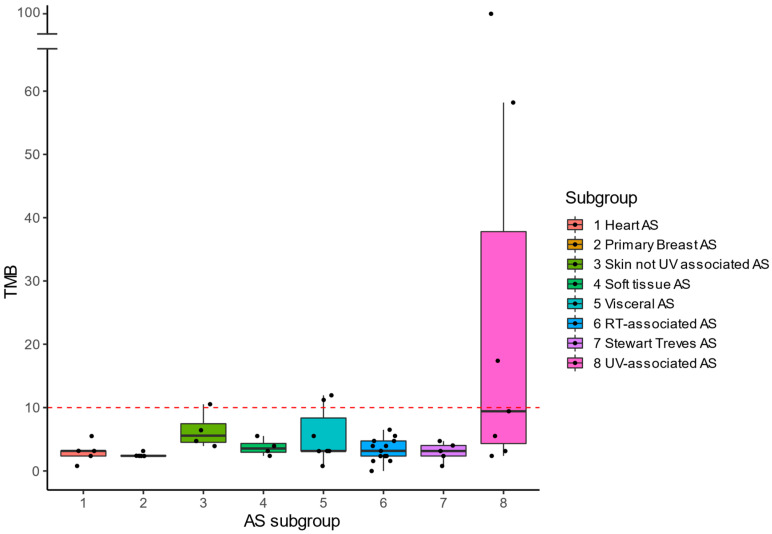
Median TMB per AS subgroup. High TMB (≥10 mut/Mb) was detected in 6 tumors (12%) in three subgroups: 3 UV associated AS, 2 Visceral AS and 1 not UV associated skin AS.

Genomic analysis was performed on tumor DNA from a subgroup of 50 patients (25 pAS and 25 sAS). Median TMB was 3.2 mut/Mb (range 0.8–11.9) in pAS vs. 3.9 mut/Mb (range 0.0–99.6) in sAS (*p* = 0.572). Figure 2 shows the median TMB per subgroup. TMB High (TMB-H; ≥10 mut/Mb) was found in 6 tumors (12%) divided over three subgroups, i.e., UV-AS (*n* = 3/7; median TMB 9.4 mut/Mb), visceral AS (*n* = 2/7; median TMB 3.2 mut/Mb) and primary skin AS (*n* = 1/4; median TMB 5.6 mut/Mb). Visceral AS with TMB-H were located in the adrenal gland and liver. None of the 50 tumors showed MSI.

Mutational signature analysis was performed for tumors with TMB-H (Figure S1). Single-base substitution signatures 7a (SBS7a) and SBS7b, associated with UV damage were detected in three tumors, all UV-AS. In one visceral AS an SBS1 signature was detected, associated with endogenous mutational processes initiated by deamination of 5-methylcytosine to thymine and generated over time. No clear mutational signature was found in the patient classified as a not UV associated skin AS.

**Figure 3:**
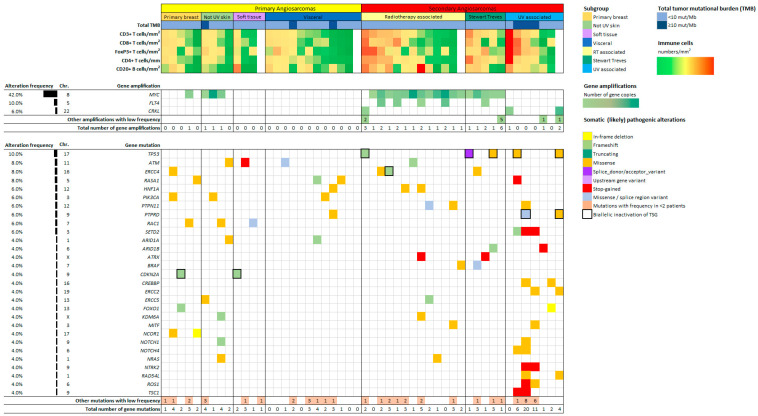
Genomic and immunological landscape of 50 angiosarcoma tumor samples. (Likely) Pathogenic mutations and gene amplifications detected in ≥2 tumors are reflected in this figure. Cell densities for CD3^+^, CD8^+^, FoxP3^+^, CD4^+^ T cells and CD20^+^ B cells are reflected based on the number of cells/mm^2^.

A (likely) pathogenic mutation or gene amplification was identified in 80% of pAS vs. 100% of sAS (*p* = 0.110). Figure 3 depicts the genetic alterations that occurred within more than one tumor sample.

Figure S2 provides a full overview of all (likely) pathogenic alterations (class 4/5). At least one (likely) pathogenic mutation was detected in 80% of the pAS vs. 88% of the sAS, (*p* = 0.702). The most frequently mutated gene was *TP53*, exclusively identified in sAS (20%, (*p* = 0.025)). Biallelic inactivation of the Tumor Suppressor Gene (TSG) was seen in all TP53 mutated cases. Other frequently found mutated genes were *ERCC4* (8%), *ATM* (8%), *RASA1* (8%), *HNF1A* (6%), *PIK3CA* (6%), *PTPN11* (6%), *RAC1* (6%) and *SETD2* (6%). *SETD2* mutations were discovered in 43% of UV-AS, but in no other AS subtype. Mutations in the DNA damage response (DDR) pathway were encountered in 24% of pAS vs. 60% of sAS (*p* = 0.021), with affection of the following genes: *ATM*, *ATRX*, *BRIP1*, *CHEK2*, *ERCC2*, *ERCC3*, *ERCC4*, *ERCC5*, *FANCF*, *FANCI, MSH2*, *MSH3*, *TP53* and *XRCC2*. DDR mutations were found in 54% of RT-AS, 57% of UV-AS and 80% of Stewart Treves AS.

Amplifications were detected in 16% of pAS vs. 84% of sAS (*p* ≤ 0.01). *MYC* amplifications being the most frequent, were found in 16% of pAS vs. 68% of sAS (*p* ≤ 0.01) with 100% of the Stewart Treves AS, 92% of the RT-AS, 75% of the not UV associated skin AS and 20% of the primary breast AS. *MYC* amplifications were not discovered in any of the pAS located in the heart, soft tissue or visceral organs nor in UV-AS. *FLT4* was amplified only in sAS (20%, *p* = 0.025)) and were seen in 31% of RT-AS, always in combination with a *MYC* amplification. *CRKL* amplifications were present in 12% of sAS (8% of RT-AS and 14% UV-AS) and none of the pAS.

Figure S3 shows all identified mutations that are classified as variant of unknown significance (class 3). In all tumor samples at least one class 3 mutation was found. Mutations in *MAGI2* (20%), *ZFHX3* (20%), *TET1* (18%), *FAT1* (16%), *FLT4* (16%), *ICOSLG* (16%) and *LRP1B* (16%) were most often present.

### 3.3. Combined Immunological and Genomic Profiles

Combined immunological and genomic data were available for 47 tumor samples (Figure 3). These samples showed similar lymphocyte cell densities compared to the entire cohort. Tumors with a DDR mutation (*n* = 21, 42%) had significantly higher cell densities for all lymphocyte subsets compared to AS without a DDR mutation including CD3^+^ (*p* = 0.004), CD8^+^ (*p* = 0.025), FoxP3^+^ (*p* = 0.010) and CD4^+^ (*p* = 0.004) T-cells and CD20^+^ B-cells (*p* = 0.031). Cell densities for all TILs were comparable in AS with and without *MYC* amplification.

Immunological and genomic data were analyzed for AS subgroups. UV-AS show the highest density for all T-cell subsets and the highest median TMB of all AS. TMB-H sAS were exclusively UV-AS. A (likely) pathogenic mutation or amplification was seen in 86% of the UV-AS. Stewart Treves AS represent the group with the second highest CD3^+^ and CD4^+^ T-cell density and the highest CD20^+^ B-cell density. A (likely) pathogenic alteration was present in 100% of this subgroup and 80% of the cases had a DDR pathway mutation. Of all pAS, visceral AS showed the highest cell density for all TILs and the highest TMB. A (likely) pathogenic mutation was found in 4 out of 7 (57%) visceral AS. A DDR pathway mutation was identified in one visceral AS (14%). The subgroup with the lowest lymphocyte density were AS of the heart. In 60% (*n* = 3) of these tumors a (likely) pathogenic mutation was found. A DDR pathway mutation was identified in one of them (20%).

## 4. Discussion

In this study we showed the heterogeneity of immunological and genomic profiles in pAS and sAS subgroups. Especially in sAS, the high immune cell density, high TMB and presence of DDR gene mutations suggest possible responsiveness to ICI. Both in pAS and sAS a high frequency of (likely) pathogenic mutations and gene amplifications were identified, with patterns that could potentially be used in boosting strategies to stimulate ICI response.

One of the main findings was a significantly higher T-cell density in sAS compared to pAS. A high T-cell density was in particular found in UV-AS, and Stewart Treves AS and to a lesser extent in RT-AS. T-cell rich TME, especially by means of CD8^+^ T-cells, has been reported as a favorable prognostic factor with regard to survival outcomes in other high grade sarcomas [33]. High CD8^+^ T-cell density has also been correlated with improved ORR to ICI in several sarcoma subtypes including AS [18]. Interestingly, cell densities of FoxP3^+^ T-cells, known for their possible immunosuppressive role within the TME, were also significantly higher in sAS compared to pAS in our study. No difference was found in B-cell infiltration between pAS and sAS. This could be explained by the relatively high B-cell density in visceral AS. With its high density of T- and B-cells, visceral AS seem to represent a specific subgroup with an immunological profile that could render them more susceptible to immunotherapy compared to other pAS.

TMB-H was present in six tumors (12%), including three UV-AS (50%), two visceral AS (33%) and one not UV associated skin AS (17%). This is in line with earlier studies showing TMB-H in especially UV-AS [16,25,26,34]. Espejo-Freire reported TMB-H in 26% of AS (total *n* = 143), predominantly of the Head and Neck (H/N) (63%) [26]. One could argue that at least a subset of their cases are UV associated as expected for this anatomic area. Visceral AS showed the second highest TMB of our AS subgroups. This finding is also underpinned by the results of Espejo-Freire et al. showing TMB-H in 14% of visceral AS. Their and our data support the hypothesis that UV-associated and visceral AS may in particular benefit from ICI treatment.

The distinction between UV-AS and non-UV associated AS of the skin is based on tumor location. Interestingly, our TMB-H UV-AS showed mutational signatures with a clear pattern associated with UV damage [30,35]. In contrast, no UV associated signature was found in the skin not-UV associated tumor with TMB-H. In half of the tumors classified as UV-AS, no TMB-H was detected. Although only a small number of AS had sufficient mutations to perform mutational signature analysis, these signatures support the clinical relevance of defining pAS and sAS in the skin. Interestingly, Chan et al. also identified more mutated and UV driven profiles in approximately half the H/N AS (*n* = 35). The others showed more mutationally quiet tumors with low TMB [34]. Weidema et al. demonstrated two distinct clusters within 11 UV-AS, using DNA methylation profiling [36]. The use of mutational signatures has also been described in the literature as a potential biomarker for response to ICI [37]. Subsequent research needs to assess the possible prognostic or predictive role of mutational signatures in AS.

In our study (likely) pathogenic mutations were identified in up to 84% of the tested tumors. Many of these mutations are druggable targets, e.g., *BRAF*, *CHEK2*, *PIK3CA*, *NRAS*, *EGFR*, *ATM*, *CDKN2A* and *RAD54L* mutations.

*PIK3CA* mutations were present in three pAS (breast, skin, visceral) (6% of tumors). The same frequency has been reported by Espejo-Freire et al. and Rosenbaum et al., while Painter et al. demonstrated with 21% a more frequent occurrence [16,25,26]. Interestingly, all tumors harboring a PIK3CA mutation in these studies were also pAS and almost exclusively located in the breast.

The DDR pathway is known to induce genomic instability and tumor evolution. DDR inhibition has shown to increase TMB and upregulate PD-L1 expression [38,39]. It also affects interaction between cancer cells and the host immune system and is associated with an activated immune microenvironment [40]. We found DDR gene mutations in 24% of pAS and 60% of sAS. Tumors with a DDR mutation had significantly higher cell densities for all lymphocyte subsets compared to AS without a DDR mutation. Teo et al. showed the association between DDR alterations and ICI treatment response in urothelial cell carcinoma patients [41]. Several clinical trials are currently investigating combination treatment with ICI and DDR targeted therapy drugs such as PARP, CDK4/6, ATR and WEE1-inhibitors [42].

*MYC* amplifications provide another interesting opportunity for boosting ICI response [43]. MYC proteins are associated with tumorigenesis and therapeutic resistance through gene amplification, translocation and mRNA upregulation. They are known to remodulate the tumor microenvironment, creating resistance to ICI [43]. Han et al. showed that MYC inhibition upregulated PD-L1 expression on tumor cells and increased the number of CD3^+^, CD4^+^, and CD8^+^ T-cells, thereby sensitizing otherwise refractory tumors for ICI treatment [43]. Several studies have shown that combination therapy with an anti PD-1 agent and a drug inhibiting the MYC pathway could be an interesting strategy to boost ICI response. The CDK7 inhibitor THZ1 in combination with ICI showed promising results in treatment for non-small cell lung cancer [44]. THZ1 inhibits MYC transcriptional activity through downregulating the p38α pathway. In our study, *MYC* was amplified in 68% of sAS. *MYC* amplifications were not exclusively present in RT-AS, but also in other AS subgroups. Interestingly, none of the UV-AS showed a *MYC* amplification, which is in concordance with previously reported studies [25,26]. For all *MYC* amplified pAS and sAS, therapeutic strategies based on MYC inhibition warrant further investigation.

*PD*-L1 status has been investigated before in AS subtypes. High PD-L1 and PD-1 expression were predominantly shown in UV-associated, visceral, and soft tissue AS. RT-AS showed predominantly high PD-L1 expression [45]. The value of PD-L1 and PD-1 expression as predictive biomarker of response to immunotherapy is limited as has been discussed extensively before [46].

Although ICI treatment is currently not registered for AS, several clinical trials are being conducted to evaluate their efficacy in AS patients. Table 4 depicts the main ongoing clinical trials that include AS patients. Where NCT05026736 includes both pAS and sAS for treatment with sintilimab, NCT04873375 inclusion is limited to sAS for treatment with cemiplimab. Other trials are evaluating combination treatments with ICI and targeted therapy or chemotherapy.

Our study has some limitations. Angiosarcomas are considered a heterogeneous disease, for which multiple classifications exist. The WHO classification does not define pAS and sAS, nor clinical subgroups [47]. Clinical subgroups have been defined in various ways previously, with pAS and sAS used most based on tumor etiology [8,25,26,45]. We used the same classification as we did before [8]. For a rare and heterogenous disease, subgroups do matter. For example, T-cell densities were high in the visceral group but not in the heart subgroup. Taken together (Table S1) this difference is not recognized. In clinics, heart AS represent an AS subtype with an aggressive behavior and less treatment opportunities. Specific clinical subgroups harbor only small numbers of patients, limiting our ability to draw definite conclusions. Second, for analyzing the TME of this large set of tumor samples, we used TMAs generated from tumor cores. Tumor cores may only represent a specific area within a heterogeneous tumor but were expected to better predict responsiveness to ICI than superficial infiltration within the edges of a tumor, especially since the detection of a tumor border in typically infiltrative growing AS is difficult. Furthermore, our data provide an insight in the TME of AS, but a clear cutoff value for using specific TILS as a (predictive) biomarker has not been established, complicating its use in clinical practice. Finally, one could remark that the TSO500 panel is a targeted NGS assay that does not comprise all genes. It does however include the vast majority of druggable cancer related genes, providing a clear overview for potential treatment options.

## 5. Conclusions

We showed a clear distinction in immunological and genomic profiles between pAS and sAS, with a T-cell infiltrated TME and frequent DDR gene mutations especially in sAS. Given the heterogeneity of angiosarcomas and the observed differences even between sAS subsets, clinical trials are needed to investigate the potential of immunotherapy.

## Figures and Tables

**Table 1:** Tumor location for the specific AS subgroups for both primary and secondary AS.

Primary AS	Location Total Group (*n* = 79)	Location TSO Selection (*n* = 25)
Soft tissue	Leg (*n* = 3) Neck (*n* = 1) Face (*n* = 1) Bottom (*n* = 1) Retroperitoneal (*n* = 1) Ureter (*n* = 1) Abdomen (*n* = 1) Mediastinum (*n* = 1) Lumbar region (*n* = 1)	Leg (*n* = 1) Face (*n* = 1) Retroperitoneal (*n* = 1) Mediastinum (*n* = 1)
Primary breast	Mamma (*n* = 20)	Mamma (*n* = 5)
Not UV associated skin	Leg (*n* = 10) Abdomen (*n* = 2) Foot (*n* = 1) Thorax (*n* = 1) Unknown (*n* = 1)	Leg (*n* = 4)
Visceral	Liver (*n* = 7) Intestine (*n* = 6) Spleen (*n* = 5) Kidney (*n* = 3) Adrenal gland (*n* = 1) Thyroid (*n* = 4) Stomach (*n* = 1) Pleura (*n* = 1)	Thyroid (*n* = 2) Kidney (*n* = 1) Adrenal gland (*n* = 1) Liver (*n* = 1) Stomach (*n* = 1) Spleen (*n* = 1)
Heart	Heart (*n* = 4) Aorta (*n* = 1)	Heart (*n* = 4) Aorta (*n* = 1)
**Secondary AS**	**Location Total Group (*n* = 178)**	**Location TSO Selection (*n* = 25)**
RT-associated	Mamma (*n* = 111) Thorax (*n* = 5) Abdomen (*n* = 3) Scalp (*n* = 2) Arm (*n* = 2) Peri-anal (*n* = 1) Bladder (*n* = 1) Shoulder (*n* = 1)	Mamma (*n* = 13)
UV associated	Scalp (*n* = 27) Face (*n* = 10) Neck (*n* = 1)	Scalp (*n* = 5) Face (*n* = 1) Neck (*n* = 1)
Stewart Treves	Arm (*n* = 11) Leg (*n* = 2) Mamma (*n* = 1)	Arm (*n* = 4) Mamma (*n* = 1)

**Table 2:** Immune cell infiltrates are depicted for primary and secondary AS. Cell densities are reflected in cells/mm^2^ with the interquartile range (IQR) for all individual immune cell subclasses.

Median Cells/mm^2^ (Interquartile Range)	Primary AS (*n* = 79)	Secondary AS (*n* = 178)	*p*-Value
CD3^+^ T-cells	245 (342)	456 (758)	*p* < 0.001
CD8^+^ T-cells	84 (129)	111 (217)	*p* = 0.033
FoxP3^+^ T-cells	22 (32)	43(95)	*p* < 0.001
CD4^+^ T-cells	127 (145)	247 (470)	*p* < 0.001
CD20^+^ B-cells	22 (73)	32 (121)	*p =* 0.417

**Table 3:** Immune densities for all AS subgroups. Cell densities are reflected in cells/mm^2^ with the IQR for all individual immune cell subclasses.

	Primary AS (*n* = 79)					Secondary AS (*n* = 178)		
Angiosarcoma Subgroup	Soft Tissue *n* = 11	Breast *n* = 20	Skin Not UV *n* = 15	Visceral *n* = 28	Heart *n* = 5	RT-Associated *n* = 126	UV Associated *n* = 38	Stewart Treves *n* = 14
Median Cells/mm^2^ (IQR)								
CD3^+^ T-cells	245 (549)	234 (246)	232 (280)	362 (440)	110 (204)	355 (605)	817 (862)	588 (734)
CD8^+^ T-cells	99 (406)	78 (94)	74 (60)	140 (194)	15 (71)	101 (186)	184 (230)	87 (209)
FoxP3^+^ T-cells	28 (63)	14 (35)	32 (50)	14 (25)	7 (27)	32 (60)	92 (161)	23 (35)
CD4^+^ T-cells	94 (137)	127 (118)	145 (143)	150 (246)	88 (107)	190 (355)	461 (460)	457 (608)
CD20^+^ B-cells	26 (82)	22 (38)	15 (11)	67 (139)	12 (34)	21 (98)	44 (145)	88 (130)

**Table 4:** Main ongoing clinical trials evaluating ICI in Angiosarcoma patients.

Study NCT Registry Number	Agent	Study Population	Phase	Recruitment Status
NCT03277924	Nivolumab + Sunitinib	Soft tissue and bone sarcomas	I/II	Active, recruiting
NCT03138161	Nivolumab + Ipilimumab + Trabectidin	Soft tissue sarcomas (including angiosarcomas)	I/II	Active, recruiting
NCT04873375	Cemiplimab	Secondary Angiosarcomas	II	Active, recruiting
NCT05026736	Sintilimab	Angiosarcomas	II	Active, recruiting
NCT04784247	Pembrolizumab + Lenvatinib	Soft tissue and bone sarcomas (including angiosarcomas)	II	Active, recruiting
NCT03512834	Avelumab + Paclitaxel	Angiosarcomas	II	Active, recruiting
NCT04339738	Nivolumab + paclitaxel; Nivolumab + cabozantinib	Skin and visceral angiosarcoma	II	Active, recruiting
NCT04551430	Nivolumab + Ipilimumab + Cabozantinib	Soft tissue sarcomas (including angiosarcomas)	II	Active, recruiting
NCT03069378	Pembrolizumab + Talimogene Laherparepvec (T-VEC)	Soft tissue sarcomas (including cutaneous angiosarcomas)	II	Active, recruiting
NCT04668300	Durvalumab + Oleclumab	Soft tissue sarcomas (including angiosarcomas)	II	Active, recruiting
NCT04095208	Nivolumab + Relatlimab	Soft tissue sarcomas (including angiosarcomas)	II	Active, recruiting
NCT04741438	Nivolumab + Ipilimumab	Soft tissue sarcomas (including angiosarcomas)	III	Active, recruiting
NCT02834013	Nivolumab + Ipilimumab	Rare tumors (including angiosarcomas)	III	Active, recruiting
NCT02815995	Durvalumab + Temelimumab	Soft tissue and bone sarcomas (including angiosarcomas)	II	Active, not recruiting

## Data Availability

The data that support the findings of this study are available from the corresponding author upon reasonable request.

## Supplementary Materials

The following supporting information can be downloaded at: https://www.mdpi.com/article/10.3390/cancers14235938/s1, Figure S1: Overview mutational signatures High-TMB; Figure S2: Genomic and immunological landscape of 50 angiosarcoma tumor samples. All (Likely) Pathogenic mutations and gene amplifications are reflected in this figure. Figure S3: Genomic and immunological landscape of 50 angiosarcoma tumor samples. All class 3 mutations (variants of unknown significance) are reflected in this figure. Table S1: Immune cell densities for AS subgroups.

## Abbreviations

AS	Angiosarcoma
DDR	DNA damage response
FFPE	Formalin-Fixed Paraffin Embedded
ICI	Immune Checkpoint Inhibition
IQR	Interquartile Range
mIHC	Multiplex immunohistochemistry (mIHC)
NK	Natural Killer
pAS	Primary Angiosarcoma
PD-1	Programmed Cell Death Ligand 1
PFS	Progression Free Survival
OS	Overall survival
PARP	Poly (ADP-ribose) polymerase
RT	Radiotherapy
sAS	Secondary Angiosarcoma
STS	Soft Tissue Sarcoma
TIL	Tumor Infiltrating Lymphocyte
TME	Tumor Microenvironment
TMA	Tissue microarrays
TMB	Tumor Mutational Burden
TSO500	Trusight Oncology 500
UV	Ultra-violet
