# Supplementary material for: Immunological and Genomic Analysis Reveals Clinically Relevant Distinctions between Angiosarcoma Subgroups
Source: Cancers (Basel). 2022 Nov 30;14(23):5938. doi: 10.3390/cancers14235938 (PMC9739001; doi:10.3390/cancers14235938)
Supplement: Supplementary file 1 [file cancers-14-05938-s001.zip › cancers-2025725-supplementary.pdf]

Figure S1: Overview mutational signatures High-TMB

SNVs	199	115	40	64	20	19
Cosine	0.979	0.975	0.955	0.805	0.946	0.754

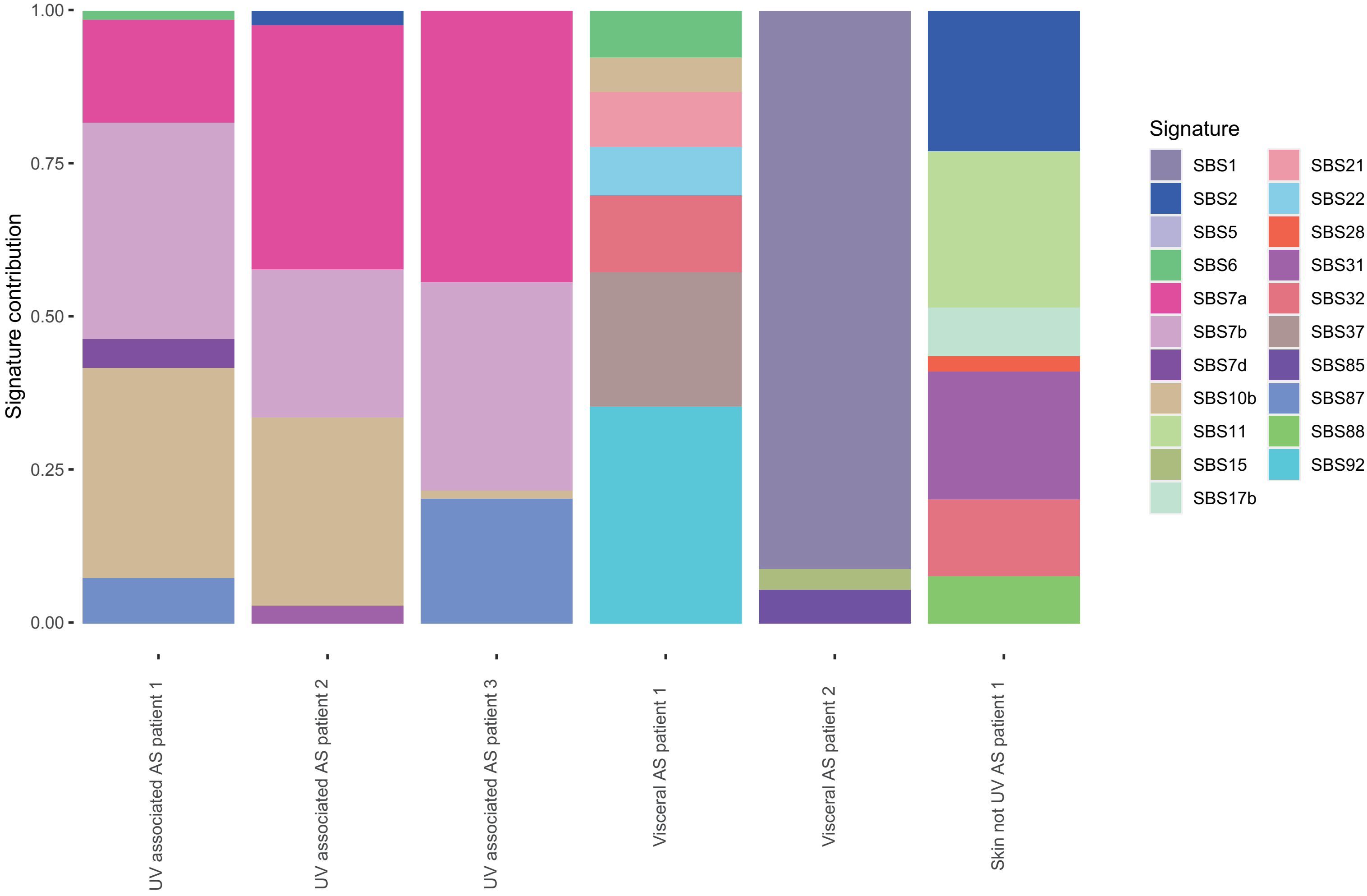

Figure S1: Mutational signatures in patients classified as high TMB (≥10 mut/Mb). Signature recognition was measured using cosine similarity. SNV; Single Nucleotide Variation.

Figure S2: Overview of all (likely) pathogenic alterations

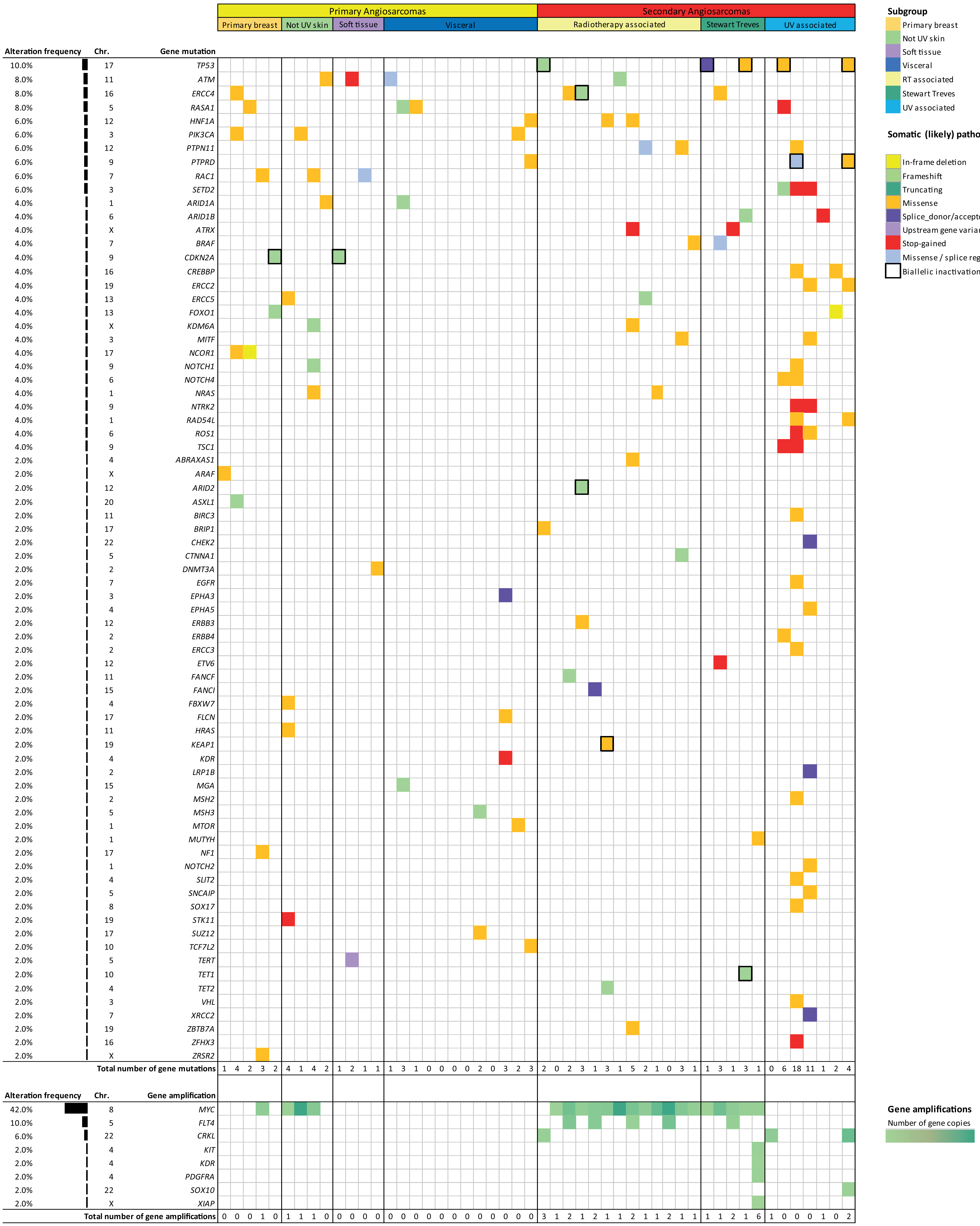

Figure S3: Overview of all class 3 mutations (variants of unknown significance)

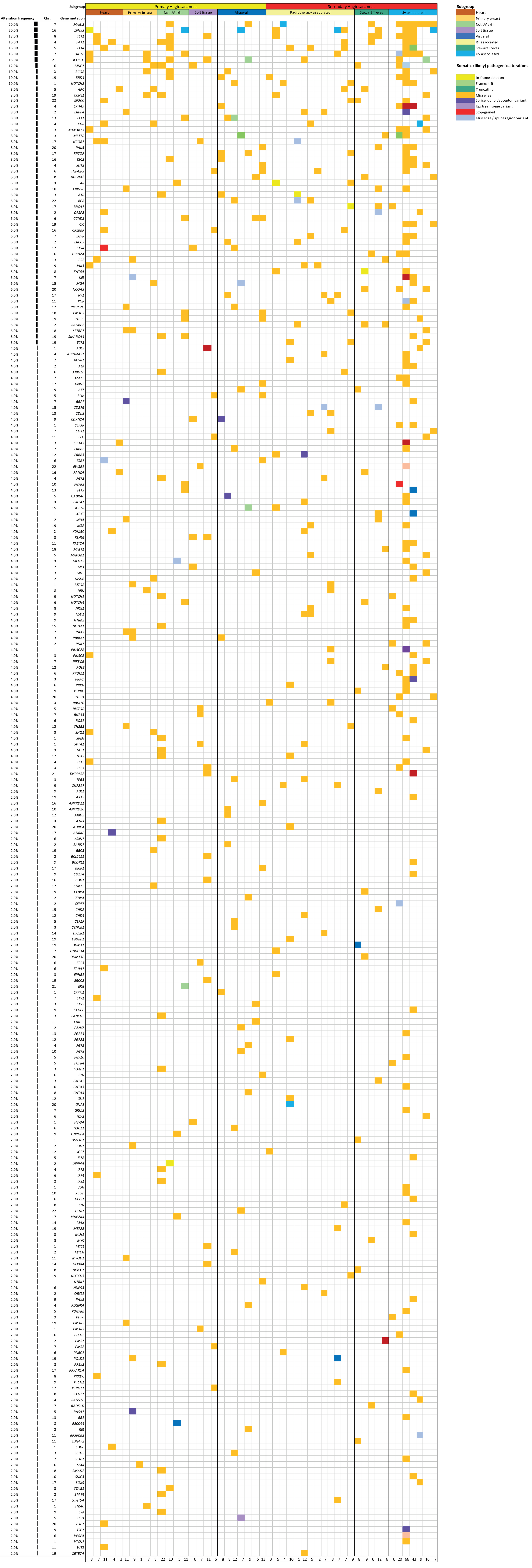

Table S1: Immune cell densities for AS subgroups. Cell densities are reflected in cells/mm^2 with the IQR for all individual immune cell subclasses. This table reflects the combined data of the visceral and hearts AS subgroup taken together in one subgroup (visceral).

	Primary AS (n=79)				Secondary AS (n=178)		
**Angiosarcoma Subgroup**	**Soft tissue n=11**	**Breast n=20**	**Skin not UV n=15**	**Visceral n=33**	**RT associated n=126**	**UV associated n=38**	**Stewart Treves n=14**
**Median cells/mm^2 (IQR)**							
CD3+ T-cells	245 (549)	234 (246)	232 (280)	336 (450)	355 (605)	817 (862)	588 (734)
CD8+ T-cells	99 (406)	78 (94)	74 (60)	109 (203)	101 (186)	184 (230)	87 (209)
FoxP3+ T-cells	28 (63)	14 (35)	32 (50)	12 (25)	32 (60)	92 (161)	23 (35)
CD4+ T-cells	94 (137)	127 (118)	145 (143)	129(237)	190 (355)	461 (460)	457 (608)
CD20+ B-cells	26 (82)	22 (38)	15 (11)	38 (122)	21 (98)	44 (145)	88 (130)
